# Identification of Predictors of Sarcopenia in Older Adults Using Machine Learning: English Longitudinal Study of Ageing

**DOI:** 10.3390/jcm13226794

**Published:** 2024-11-12

**Authors:** Nieves Pavón-Pulido, Ligia Dominguez, Jesús Damián Blasco-García, Nicola Veronese, Ana-María Lucas-Ochoa, Emiliano Fernández-Villalba, Ana-María González-Cuello, Mario Barbagallo, Maria-Trinidad Herrero

**Affiliations:** 1 Clinical and Experimental Neuroscience (NiCE), Institute for Aging Research, Biomedical Institute for Bio-Health Research of Murcia (IMIB-Arrixaca), School of Medicine, Campus Mare Nostrum, UniWell, University of Murcia, 30100 Murcia, Spain; jesusdamian.blascog@um.es (J.D.B.-G.); anamaria.lucas1@um.es (A.-M.L.-O.); emiliano.fernandez@um.es (E.F.-V.); agcuello@um.es (A.-M.G.-C.); 2 Department of Automation, Electrical Engineering and Electronic Technology, Campus Muralla del Mar, Technical University of Cartagena, 30202 Cartagena, Murcia, Spain; nieves.pavon@upct.es; 3 Geriatric Unit, Department of Medicine, University of Palermo, 90100 Palermo, Italy; ligia.dominguez@unipa.it (L.D.); nicola.veronese@unipa.it (N.V.); mario.barbagallo@unipa.it (M.B.); 4 Faculty of Medicine and Surgery, University of Enna “Kore”, 94100 Enna, Italy

**Keywords:** artificial intelligence, machine learning, decision tree, sarcopenia, English Longitudinal Study of Ageing, older adults, epidemiology, aging, cohort, prospective

## Abstract

**Background:** After its introduction in the ICD-10-CM in 2016, sarcopenia is a condition widely considered to be a medical disease with important consequences for the elderly. Considering its high prevalence in older adults and its detrimental effects on health, it is essential to identify its risk factors to inform targeted interventions. **Methods:** Taking data from wave 2 of the ELSA, using ML-based methods, this study investigates which factors are significantly associated with sarcopenia. The Minimum Redundancy Maximum Relevance algorithm has been used to allow for an optimal set of features that could predict the dependent variable. Such a feature is the input of a ML-based prediction model, trained and validated to predict the risk of developing or not developing a disease. **Results:** The presented methods are suitable to identify the risk of acquired sarcopenia. Age and other relevant features related with dementia and musculoskeletal conditions agree with previous knowledge about sarcopenia. The present classifier has an excellent performance since the “true positive rate” is 0.81 and the low “false positive rate” is 0.26. **Conclusions:** There is a high prevalence of sarcopenia in elderly people, with age and the presence of dementia and musculoskeletal conditions being strong predictors. The new proposed approach paves the path to test the prediction of the incidence of sarcopenia in older adults.

## 1. Introduction

Sarcopenia traditionally refers to “age-related muscle loss, affecting a combination of appendicular muscle mass, muscle strength, and/or physical performance measures” [1,2]. This condition is now widely considered to be a medical disease after its introduction in the ICD-10-CM in 2016 [3]. Even though it is generally studied in geriatrics, it is still underdiagnosed, since there is no real consensus on how it should be diagnosed [4]. The prevalence of sarcopenia is still discussed. A recent meta-analysis including only community-dwelling adults aged ≥ 55 years reported a prevalence of at least 10%, but it depends on the method used for assessing sarcopenia [5]. Sarcopenia may have important consequences for older people. For example, highly suggestive evidence supports the idea that sarcopenia is associated with the risk of mortality, disability, and falls, as well as relevant outcomes for older people [6]. Therefore, sarcopenia is associated with significant health care costs [6]. Considering the high prevalence of sarcopenia in older adults, the adverse effects on health, and the significant health care costs, it is important to identify the risk factors of sarcopenia in older adults to achieve targeted interventions.

Artificial intelligence (AI) is the term employed to describe the “use of computers and technology to simulate intelligent behavior and critical thinking comparable to a human being” [7]. In this regard, AI may help with a better identification of factors associated with a medical condition (e.g., sarcopenia) using complex databases and big-data information. The studies regarding AI and sarcopenia are mainly focused on how to diagnose sarcopenia using this tool, particularly for the assessment of body composition [8]. On the contrary, only one study explored the risk factors potentially associated with sarcopenia using artificial intelligence; this should be explored further, as risk factors are usually interrelated and only the use of conventional statistical methods could complicate the detection of sarcopenia [9].

Given this background, the aim of the present study was to investigate which factors were significantly associated with sarcopenia using artificial intelligence in a large representative sample population of English older adults.

## 2. Materials and Methods

### 2.1. Study Population

This study used data from wave 2 of the English Longitudinal Study of Ageing (ELSA), a prospective and nationally representative cohort of men and women living in England [10]. Wave 2 (baseline survey) was conducted between 2004 and 2005. The ELSA was approved by the London Multicentre Research Ethics Committee (MREC/01/2/91). Written informed consent was obtained from all participants.

### 2.2. Sarcopenia (Dependent Variable)

Following the criteria of the Revised European Consensus on the definition and diagnosis of sarcopenia [11,12], this condition was defined as having weak handgrip strength (defined as <27 kg for men and <16 kg for women using the average value of three handgrip measurements of the dominant hand) [11] and low skeletal muscle mass (SMM), as reflected by the lower skeletal mass index (SMI). SMM was calculated based on the equation proposed by Lee and colleagues [13]: SMM = 0.244 × weight + 7.8 × height + 6.6 × sex − 0.098 × age + race − 3.3 (where female = 0 and male = 1; race = 0 [White and Hispanic], race = 1.9 [Black], and race = −1.6 [Asian]). SMM was further divided by body mass index (BMI), based on weight and height measured by a trained nurse, to create an SMI [14]. Low SMM was defined as the lowest quartile of the SMI, based on sex-stratified values [15].

### 2.3. Other Factors

The selection of factors (predictor variables), possibly associated with sarcopenia, was based on the literature. The following factors were included: age, sex, years of schooling, number of alcoholic drinks consumed per week, retirement age (all considered as continuous variables), CASP-19 (Control, Autonomy, Self-realization, and Pleasure) indicating quality of life [16], CES-D (Center for Epidemiologic Studies Depression Scale) indicating the presence of depressive symptoms [17], body mass index (as a continuous variable), smoking status (ever, present, never), marital status (married, widowed, other status), difficulty in activities of daily living (ADL) and in instrumental ADL, physical activity level (high, moderate, low, sedentary), and the number of comorbidities. All of the factors are extensively reported in Appendix A.

### 2.4. Methods and Tools Used for Selecting Features and Obtaining a Prediction Model

MATLAB 2022 (the programming and numeric computing platform) was used as the tool for carrying out all of the experiments. The Statistics and Machine Learning Toolbox 2022 was selected, since it allows for multidimensional data analysis and feature extraction to be carried out, providing dimensionality reduction and feature selection methods to identify those variables with maximum predictive power. Furthermore, the toolbox provided supervised, semi-supervised, and unsupervised algorithms based on machine learning (ML), including boosted decision trees, ready to be programmed, configured, and applied over raw data in multiple formats. The database was imported from an Excel 2022-formatted file. The following steps were carried out to select a collection of relevant features, which had been used as inputs in an ML-based prediction model: selection of relevant features, statistical tests for demonstrating that such features allow for the generation of a prediction model with at least the same accuracy than the one including all of the features, and the training and validation of the model.

#### 2.4.1. Selection of Relevant Features

The fscmrmr MATLAB function was selected in the present study. The function implements the Minimum Redundancy Maximum Relevance (MRMR) algorithm [18], which allowed features to be ranked for classification. It found an optimal set of features that was mutually and maximally dissimilar and could represent the response variable effectively. The algorithm minimized the redundancy and maximized the relevance of a feature set to the response variable. It quantified the redundancy and relevance using the mutual information on variables pairwise, mutual information on features, and mutual information on a feature and its response. It was useful for application in a previous preprocessing stage and in the classification problems.

The goal of the MRMR algorithm was finding an optimal collection of predictors *C* that maximized the relevance and minimized the redundancy of *C*, denoted as *W_C_*, where

(1) $$V_{C}=\frac{1}{\left|C\right|}\sum_{x\epsilon C}I\left(x,y\right)$$

(2) $$W_{C}=\frac{1}{{\left|C\right|}^{2}}\sum_{x,z\epsilon C}I\left(x,z\right)$$

*I* is the mutual information between two variables that measured how much the uncertainty of one variable could be reduced when knowing the other variable:

(3) $$I\left(X,Z\right)=\sum_{i,j}P\left(X=x_{i},\;Z=z_{j}\right)\log\frac{P\left(X=x_{i},\;Z=z_{j}\right)}{P\left(X=x_{i}\right)P\left(Z=z_{j}\right)}$$

*X* and *Z* are two discrete random variables. If *X* and *Z* are independent, *I* is 0. If *X* and *Z* are the same random variables, they are the entropy of X. The fscmrmr MATLAB function uses the concept of mutual information to compute such a value for both categorical and continuous (numerical) variables, discretizing numerical ones into 256 bins or the number of unique values in the variable if it was less than 256. It also found optimal bivariate bins for each pair of features using the adaptive algorithm described in [19].

The fscmrmr function allowed for the missing values to be considered for ranking features.

#### 2.4.2. Prediction Model

The database was split into two tables, training (2995 samples) and validation (1999), meaning that 40% of the samples were reserved for validation. As a previous step before training the selected model, a test was carried out to prove that training a model with only the features selected using the MRMR algorithm, compared to training it with all the features included in the database, does not lose accuracy. The MATLAB function testckfold was used for this purpose, since it statistically assesses the accuracies of two classification models by repeatedly cross-validating such models, calculating the differences in the classification loss and formulating the test statistics by combining the classification loss differences. The function returned a logical value of 0 with a specific *p*-value, if rejecting the null hypothesis failed. The null hypothesis was that the model with all of the predictors was, at most, as accurate as the model with the selected ones.

Finally, a ML-based model for prediction purposes was selected, in particular, RUSBoosted Trees, implemented in MATLAB using the function fitcensemble, passing the method ‘RUSBoost’ to it as a parameter. Random under-sampling boosting (RUSBoost) was especially effective at classifying imbalanced data, that is, when a class had much fewer members than the another. In these cases, the unbalance was evident, such as in the case of 4994 samples, where only 460 were individuals diagnosed with sarcopenia. Therefore, class 1 (healthy individuals) contained 4534 observations and class 2 (individuals with sarcopenia) involved 460 observations. The RUS (Random Under Sampling) algorithm took N, the number of members in the class with the fewest members in the training data, as the basic unit for sampling; then, the classes with more members were under sampled by taking only N observations of every class. In this case, there were 2 classes; then, for each weak learner in the ensemble, RUSBoost took a subset of the data with N observations from each of the 2 classes.

## 3. Results

Among the 9432 participants present at wave two, 3186 were excluded since they aged less than 60 years, 1157 since no information regarding weight or height was available, and 95 as no information regarding handgrip strength was present. Therefore, 4994 participants were included in the analysis. The mean age of the participants was 70.9± SD 7.7 years (range: 60–90), and 45.3% were men. The overall prevalence of sarcopenia was 9.2% (95%CI: 8.4–10.0%) with a significant higher presence of this condition in women than in men (10.2 vs. 8%, *p* = 0.001).

**Figure 1:**
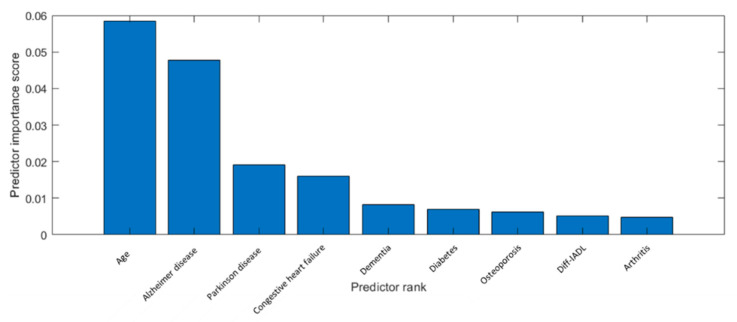
List of the nine best features ranked using the MRMR-based method.

Only the nine best ranked features were selected because the model trained offered the best results in terms of accuracy with those features. Figure 1 shows the nine best features ranked using the MRMR-based algorithm. 

Two training datasets, one including all of the forty-seven features and another one only including the selected nine features described in Table 1, were used as inputs when the “testckfold” function was applied. Their outputs proved the null hypothesis that the model with all of the predictors was, at most, as accurate as the model with the nine ones. In this case, the output of the function was 0, indicating the failure to reject the null hypothesis, with a *p*-value of 0.9918. This could be interpreted as meaning that, when using the model with the nine predictors, it did not result in loss of accuracy compared to the model with all of the predictors.

**Figure 2:**
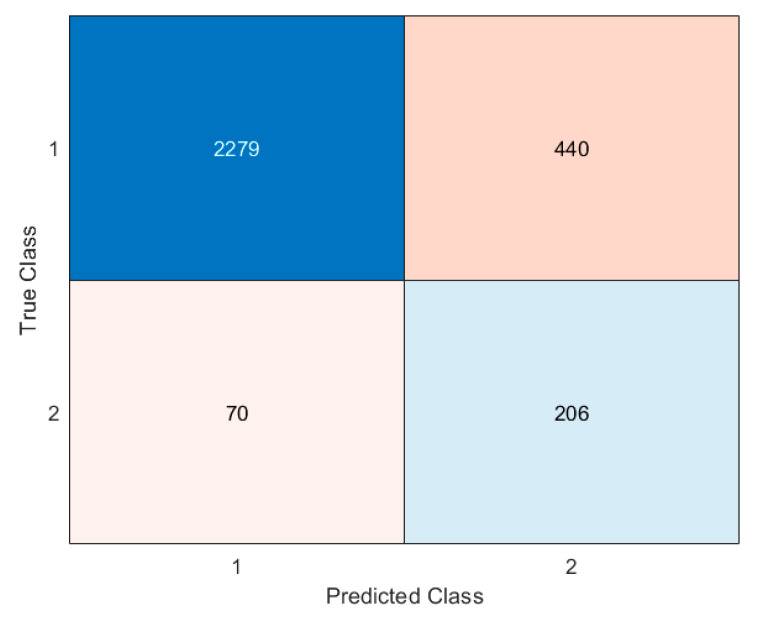
Confusion matrix for the training dataset.

**Figure 3:**
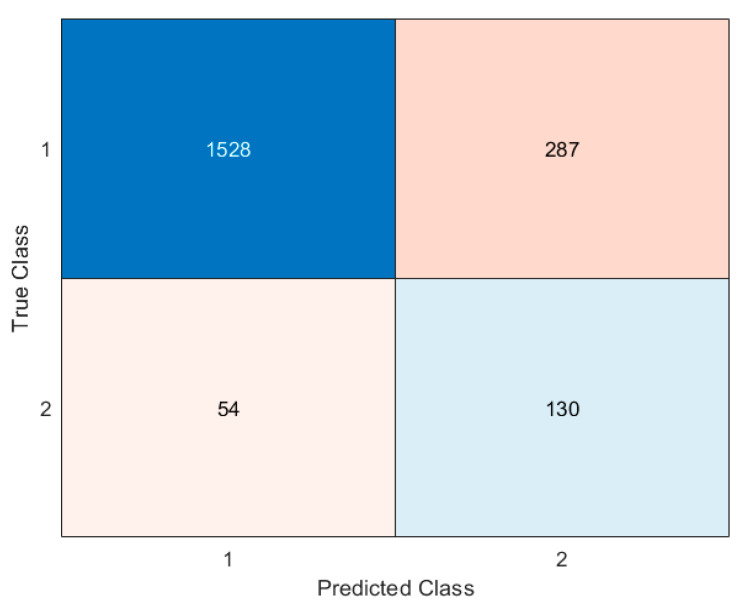
Confusion matrix for the test dataset.

Figure 2 and Figure 3 show the results obtained after validating the model with the training and test datasets. Figure 2 shows the performance of the model when it was applied using data from the training dataset. Figure 3 offers the performance of the model when it was applied using data from the validation dataset.

**Figure 4:**
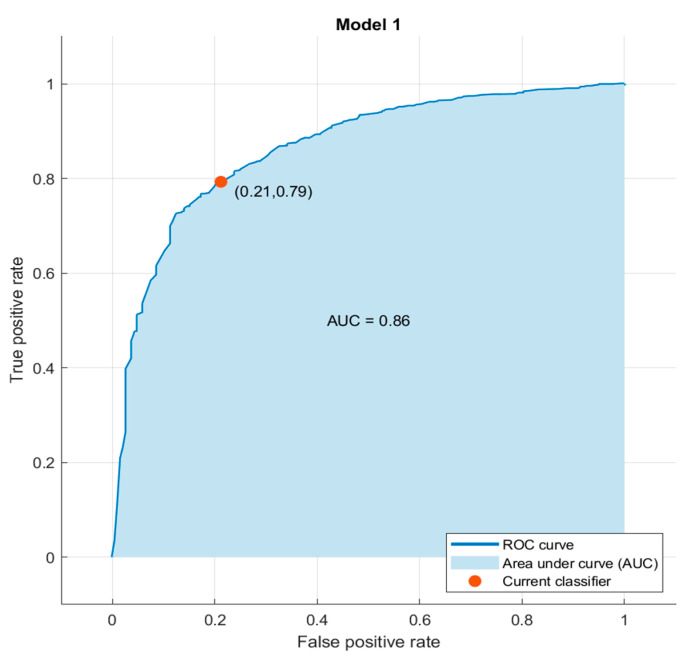
ROC curve.

Figure 4 demonstrates the ROC (Receiving Operating Characteristic) curve, which allows for the true positive rate (TPR) against the false positive rate (FPR) to be represented. The ROC curve obtained after testing the model demonstrates that the attained classifier shows a good behavior because the “true positive rate” was high (0.81) and the “false positive rate” was moderately low (0.26). Then, the model could be appropriate for applying automatic initial screening of potential patients.

## 4. Discussion

In this work, which included about 5000 elderly participants, we found that the prevalence of sarcopenia was high, affecting 1/10 elderly people. Using an ML-based approach, the main factors associated with sarcopenia were age and the presence of dementia. Of the other seven remaining factors included, we found that Parkinson’s Disease, congestive heart failure, diabetic eye disease, osteoporosis, disability in IADL, and arthritis were also significantly associated, although to a lesser extent, with the risk of sarcopenia.

We observed that sarcopenia is a common condition in the ELSA study, since about one person out of ten was affected. These data were in line with other epidemiological works, even if great differences were present due to the different definitions and settings in which these studies were conducted [20]. Moreover, among the several available factors present in the ELSA study, only a few were associated with a higher presence of sarcopenia. In particular, dementia (especially Alzheimer’s disease) was significantly present in sarcopenia, supported by other studies. For example, in a systematic review of 13 studies, the authors found a higher prevalence of sarcopenia in participants with dementia compared to controls [21]. This epidemiological evidence could be confirmed by several justifications, including that people having dementia were commonly more sedentary than cognitively intact counterparts [22]. Moreover, muscle mass loss has been associated with Alzheimer’s disease related to brain atrophy, indicating that brain atrophy and loss of muscle mass may co-occur [23]. In addition, our study suggests that sarcopenia could be strongly associated with some musculoskeletal conditions such as osteoporosis or arthritis. Our findings are supported by the previous literature [24,25]. It is also worth highlighting that sarcopenia was associated with the presence of congestive heart failure; this association between sarcopenia and congestive heart failure seems to start at a younger age, differently from the conditions above [26].

The use of ML-based techniques for extracting relevant features from the dataset confirmed several of the mentioned assumptions. This is a novel finding, since the data themselves include knowledge that has stayed hidden from the human eye, but it could be extracted by applying the suitable algorithms. In this work, we obtained a set of relevant features by applying the MRMR algorithm [18]. That means that each selected feature was calculated as relevant according to the data, and then it had an influence on the behavior of the response variable. In the present case, the method was capable of detecting that age was relevant, agreeing with previous knowledge about sarcopenia. In addition, the MRMR method also had relevant characteristics related to dementia and musculoskeletal conditions. We also demonstrated that it was possible to generate a model for predicting Sarcopenia by only considering the extracted features with the MRMR method. As the data were not balanced and the number of healthy individuals was considerably greater than subjects with sarcopenia, the Random Under-Sampling boost (RUSboost) selected for classifying inputs, and the results were good both for training and validation data, offering an appropriate value of AUC, obtaining a high “true positive rate” (0.81) and moderately low “false positive rate” (0.26).

Decision tree models effectively handle a wide variety of types of data, including skewed data and data requiring slight pre-processing and manipulation, which differs from traditional statistical techniques like regression. These kind of models were more transparent regarding interpretation than black-box models, and this is an advantage in the field of health, since the interpretability of the model could be necessary to comprehend the mechanism of illness. In particular, decision tree algorithms are classifier models that learn from a training dataset and generate a flowchart-like tree structure illustrating the relationships between input predictors and the target class. Any specific input is classified by starting at the root of the tree, and the output (the class) is provided depending on the values of the attributes, tracing a path down through the branches of the tree. As decision trees could be unstable, since small changes in the data could generate a completely different tree, they are particularly well-suited for ensembles. Ensemble learning allows for multiple classifiers to be used and weighted and then combined for obtaining a better classifier with better performance in comparison to individual classifiers. There are different ensemble methods, and boosting is a general method for improving the performance of a weak learner (such as a decision tree).

RUSboost demonstrated itself to be specifically designed for solving this kind of problem, particularly when it found skewed data (many more observations of one class), as occurred in the sarcopenia database. Moreover, as in all of the boost algorithms, RUSboost uses shallow trees, and this construction could be better in terms of time and the usage of computational resources. Therefore, by combining the feature extraction method that generated the more relevant features in the context of predicting sarcopenia and the RUSboost model, the results were promising, even when the dataset was very unbalanced, and it was possible to retrain the system if new training data were included in the dataset, allowing for the model to be improved if new studies were made. The present findings demonstrate that machine learning allowed for knowledge to be extracted without carrying out tedious preprocessing tasks, and it offered a robust predictor in terms of true and false positives, as the ROC curve shows in Figure 4, which is useful for applying automatic initial screenings of potential patients.

The findings of our study should be interpreted within its limitations. First, the definition of sarcopenia that was made in the ELSA study using handgrip strength and an equation derived from anthropometric measures was not validated among UK people [27]. Second, the cross-sectional nature of this study may preclude any of the casual inferences of our findings. Third, the skeletal muscle mass (SMM) was calculated based on an equation, and this could be inaccurate. Finally, we did not consider, even if they were important according to the literature, several potential risk factors for sarcopenia, including information regarding diet [28].

## 5. Conclusions

The present study, conducted in the context of the ELSA cohort and using a machine learning approach, suggests that the prevalence of sarcopenia in elderly people is high. In particular, age and the presence of dementia are among the strongest predictors of sarcopenia in older people, by means of new methodology and analyses, using artificial intelligence with four different algorithms. Despite these factors being already extensively studied and included regularly in standard comprehensive geriatric assessments, this new approach opens new avenues to test predictions of the incidence of sarcopenia in older adults, a condition that is highly associated with disability and mortality.

## Figures and Tables

**Table 1:** List of ranked features with their descriptions and types.

Name	Description	Type
Age (r2agey)	Age of patient	Numerical
Alzheimer’s disease (hedibad)	Ever reported Alzheimer’s Disease (diagnosed)	Categorical
Parkinson disease (hedibpd)	Ever reported Parkinson’s Disease (diagnosed)	Categorical
Congestive heart failure (hedimhf)	Ever reported congestive heart failure (diagnosed)	Categorical
Dementia (hedibde)	Ever reported dementia or memory impairment (diagnosed)	Categorical
Diabetes (heoptdi)	Ever reported diabetic eye disease (diagnosed)	Categorical
Osteoporosis (hedibos)	Ever reported osteoporosis (diagnosed)	Categorical
Diff-IADL (r2iadlza)	r2iadlza:w2 R Some Diff-IADLs:/0–5	Categorical
Arthritis (hedibar)	Ever reported arthritis (diagnosed)	Categorical

## Data Availability

The data are contained within the article and Appendix A.

## Appendix A

**Table A1:** Potential predictors (independent variables) included in the database containing the ELSA study’s data.

Name	Description	Type
r2agey	Age of patient	Numerical
gender	Gender	Categorical
bmi	Body Mass Index (BMI)	Numerical
raedyrs_e	Years of education	Numerical
r2mstat	Marital status	Numerical
r2smokev	Smoke ever	Categorical
r2smoken	Smokes now	Categorical
r2adla	Some Diff-ADLs/0–5	Numerical
r2cesd	CESD score	Numerical
hedimbp	Ever reported high blood pressure (HBP) (diagnosed)	Categorical
hediman	Ever reported angina (diagnosed)	Categorical
hedimmi	Ever reported myocardial infarction (diagnosed)	Categorical
hedimhf	Ever reported congestive heart failure (diagnosed)	Categorical
hedimhm	Ever reported heart murmur (diagnosed)	Categorical
hedimar	Ever reported arrhythmia (diagnosed)	Categorical
hedimdi	Ever reported diabetes or high blood sugar (HBS) (diagnosed)	Categorical
hedbts	Ever reported diabetes (diagnosed)	Categorical
hedimst	Ever reported stroke (diagnosed)	Categorical
cvd7dihb	Ever reported any of 7 cvd-related diseases (excluding HBP)	Categorical
cvd7dbts	Ever reported any of 7 cvd-related diseases (excluding HBS and HBP)	Categorical
hediblu	Ever reported hedibonic lung disease (diagnosed)	Categorical
hedibas	Ever reported asthma (diagnosed)	Categorical
hedibar	Ever reported arthritis (diagnosed)	Categorical
hedibos	Ever reported osteoporosis (diagnosed)	Categorical
hedibca	Ever reported cancer (diagnosed)	Categorical
hedibpd	Ever reported Parkinson’s Disease (diagnosed)	Categorical
hedibps	Ever reported psychiatric disorder (diagnosed)	Categorical
hedibad	Ever reported Alzheimer’s Disease (diagnosed)	Categorical
hedibde	Ever reported dementia or memory impairment (diagnosed)	Categorical
heoptgl	Ever reported glaucoma (diagnosed)	Categorical
heoptdi	Ever reported diabetic eye disease (diagnosed)	Categorical
heoptmd	Ever reported macular degeneration (diagnosed)	Categorical
heoptca	Ever reported cataract (diagnosed)	Categorical
palevel	Physical activity summary	Categorical
raracem	Race—masked	Categorical
rarelig_e	Religion	Categorical
r2iadlza	r2iadlza:w2 R Some Diff-IADLs:/0–5	Categorical
r2drinkd_e	Days/week drinks	Numerical
s2readrc	Word recall list read by	Numerical
s2imrc	Immediate word recall	Numerical
s2dlrc	Delayed word recall	Numerical
s2orient	Cognition orient (summary date naming)	Numerical
s2verbf	Verbal fluency score	Numerical
s2tr20	Recall summary score	Numerical
s2prmt1	Prospective memory task 1	Numerical
r2retage	Retirement age	Numerical
CASP2	CASP-19 quality of life scale	Numerical
